# Child gigantomastia revealing juvenile giant fibroadenomas

**DOI:** 10.1259/bjrcr.20210181

**Published:** 2021-11-30

**Authors:** Lina Belkouchi, Siham El Haddad, Nidal Mrani Alaoui, Nazik Allali, Latifa Chat

**Affiliations:** 1 Department of Radiology, Children Hospital of Rabat, Ibn Sina University Hospital, Faculty of Medicine and Pharmacy of Rabat, Rabat, Morocco

## Abstract

Breast masses in children and adolescents are uncommon. They can be caused by tumors such as fibroadenomas and phyllode tumors. These masses can cause gigantomastia, due to their rapidly increasing size. We report the case of a 12- year-old patient admitted in our department for a rapidly growing gigantomastia evolving in a matter of 8 months. Imaging features were in favor of juvenile giant fibroadenomas and diagnosis was confirmed by biopsy. Juvenile giant fibroadenomas are rare and represent 0.5–2% of all fibroadenomas, their exact etiology is unknown. They affect children and adolescents, with a predominance in African-American females. They may cause breast enlargement and asymmetry. The rapid growth causes anxiety and is the main cause of consultation. These tumors although benign, have to be treated rapidly because they can destruct up to 80% of the surrounding normal breast tissue, and conservatory treatment cannot be done.

## Clinical presentation

A 12-year-old girl, without any medical history records, was admitted for a bilateral gigantomastia that had been developing for almost 8 months.

## Investigations and imaging findings

Biology tests were normal, and no hormonal imbalances were noted.

**Figure 1.**
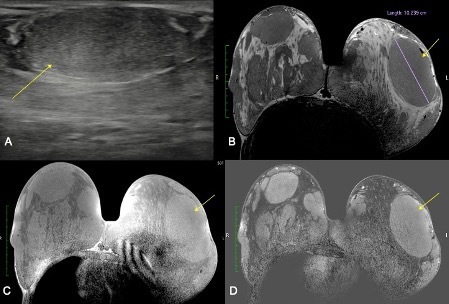
Breast ultrasound (**A**) and MRI in *T_2_* weighted images (**B**), *T_1_* weighted images (**C**), and substruction images (**D**), showing an oval shaped, well-circumscribed hypoechoic mass, with a slight posterior acoustic enhancement (yellow arrow, image A), MRI shows multiples masses, oval shaped, well-circumscribed, with intermediate T2 signal, T1 isosignal intensity, and a homogeneous contrast enhancement, with the biggest one measuring 10 cm (yellow arrow in images B, **C and D**)

A breast ultrasound objected multiple breast masses, well-circumscribed, hypoechoic, homogeneous with posterior acoustic enhancement (Figure 1A).

A breast MRI showed the presence of multiple masses, oval shaped, well-circumscribed, with intermediate signal in *T_2_* weighted sequences (Figure 1B), isosignal intensity in *T_1_* weighted sequences (Figure 1C), with a homogeneous benign contrast enhancement (Figure 1D), measuring at least 5 cm, with the biggest one on the right breast measuring 7 cm and the largest one the left breast measuring 10 cm (Figure 1B, yellow arrow), in favor of juvenile giant fibroadenomas.

## Differential diagnosis

The main differential diagnosis is phyllodes tumors. Clinical symptoms are mainly rapidly growing breast masses to which imaging features can resemble giant or juvenile fibroadenomas, with benign features. Therefore, it is sometimes difficult to differentiate between the two, and only histological confirmation allows diagnosis.

## Treatment

Surgical excision and histological examination were made, and the diagnosis of juvenile giant fibroadenomas was confirmed.

**Figure 2.**
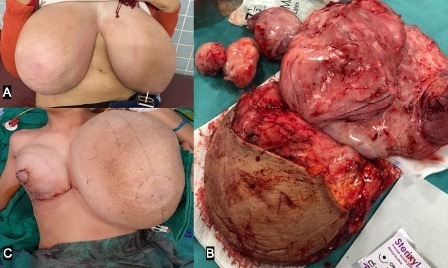
Clinical image showing bilateral gigantomastia (**A**), with multiples masses removed surgically (**B**), and right breast reconstruction (**C**)

The patient underwent surgery with removement of the masses and reconstructions (Figure 2).

## Outcome and follow-up

The patient benefited from a total removal of the masses with breast reconstruction, no mastectomy was needed, she recovered well.

## Discussion

Breast masses in pediatrics are rare and mostly benign, with a prevalence of 3.25% in adolescents between the ages of 10–21, and fibroadenomas as the most common lesions found, representing 68% of all breast masses, and 40–94% of all biopsied breast lesions.^1–3^

Adolescent breast masses classify into four common types: simple fibroadenomas, giant fibroadenomas, juvenile fibroadenomas and phyllodes tumors.^2^

While simple fibroadenoma of the breast is the most common lesion, giant juvenile fibroadenoma is a very rare variant, with an incidence of 0.5–2%, representing 7–8% of all fibroadenoma subtypes, and the most common cause of unilateral gigantomastia in young female patients.^3,4^

They are seen more frequently in African-American population,^1^ and are characterized by rapid growth causing a rapid breast enlargement.

It is called juvenile if it occurs in adolescents between the ages of 10- and 18-year-old, and it is called giant when its size exceeds 5 cm, weighs more than 500 g or replaces at least 80% of the breast.^3^ They are painless and cause rapid breast enlargement, often present as multiple bilateral lesions of the breasts. Its etiology remains unprecise, and some hormonal theories at cause are reported.^5^

Clinical presentation includes breast enlargement and asymmetry, or palpation of multiple painless masses.

Imaging has a major role in diagnosis, in children and adolescents radiological approach differs from adult females and the ultrasound is the main diagnostic examination, associated with breast MRI.^4,5^

Mammography isn’t recommended.

In ultrasonography, its typical appearance is the presence of well-circumscribed round or oval shaped masses, sometimes lobulated, with a parallel orientation, a fairly uniform hypoechoic, or anechoic with low level internal echoes, and sometimes with a posterior acoustic enhancement. In Doppler evaluation, it can be avascular or show some central vascularity.^4,5^

In MRI, they may have a variable appearance, as oval or round shaped masses, well-circumscribed or lobulated, either presenting a hypointense signal in *T_1_* weighted sequences, hyperintense signal in *T_2_* weighted sequences (usually due to hypercellularity) with enhancement,^5^ they can also appear in a low signal intensity in T2 without any enhancement. When the lesion is enhanced, it may contain some internal septations, corresponding to collagenous bands.^4^

The differential diagnosis to juvenile fibroadenomas are phyllodes tumors. In imaging, it is sometimes difficult to differentiate between the two, and that is why a diagnostic biopsy is necessary.

The safest method of biopsy is a complete surgical excision of the mass(es) to eliminate malignancy.

Juvenile fibroadenomas have a hypercellular stromal proliferation with pericanalicular or intracanalicular patterns.^4^

The histological difference between phyllodes tumors and giant juvenile fibroadenoma is the presence of leaf-like projections and stromal cells atypia.^3^

Treatment consists of a conservative approach with surgical excision of the masses, especially in rapidly growing masses of the breast in adolescents even if the biopsy is benign. However, in case of giant juvenile fibroadenomas with destruction of more than 50% of surrounding normal breast tissue, a mastectomy with reconstructions is considered and preferred.^1,2^

## Learning points

- Gigantomastia in adolescents can be either due to juvenile hypertrophy or breast masses.
- Juvenile giant fibroadenomas may be at cause and can be suspected clinically through palpable masses.
- Ultrasonography and MRI are the examination of choice for breast imaging in children and adolescents.
- Imaging shows bilateral masses > 5 cm with benign features (BIRADS 3).
- Main differential diagnosis is Phyllodes tumors that may have same clinical and radiological features; therefore, a histological confirmation is necessary.
- Clinicians and radiologists should be aware of this diagnosis for better approach and early conservative treatment.
